# Effectiveness and Safety of Tofacitinib in the Management of Ulcerative Colitis: A Brazilian Observational Multicentric Study

**DOI:** 10.1093/crocol/otac050

**Published:** 2022-12-19

**Authors:** Ramir Luan Perin, Daniela Oliveira Magro, Adriana Ribas Andrade, Marjorie Argollo, Nayara Salgado Carvalho, Adérson Omar Moura Cintra Damião, Adriana Zanoni Dotti, Sandro da Costa Ferreira, Cristina Flores, Juliano Coelho Ludvig, Rodrigo Bremer Nones, Natalia Sousa Freitas Queiroz, Rogério Serafim Parra, Flavio Steinwurz, Fabio Vieira Teixeira, Paulo Gustavo Kotze

**Affiliations:** Universidade de Passo Fundo (UPF), Passo Fundo, Brazil; Universidade Estadual de Campinas (UNICAMP), Campinas, Brazil; Universidade Estadual da Bahia (UNEB), Salvador, Brazil; Hospital São Luiz (Rede D’or), São Paulo, Brazil; Hospital São Luiz (Rede D’or), São Paulo, Brazil; Universidade de São Paulo (USP), São Paulo, Brazil; Hospital de Clínicas das UFPR, Curitiba, Brazil; Faculdade de Medicina de Ribeirão Preto da Universidade de São Paulo (FMRPUSP), Ribeirão Preto, Brazil; Instituto do Aparelho Digestivo (IAD), Porto Alegre, Brazil; Clínica ESADI, Blumenau, Brazil; Hospital Nossa Senhora das Graças, Curitiba, Brazil; Hospital Santa Cruz, Curitiba, Brazil; Hospital de Clínicas das UFPR, Curitiba, Brazil; Hospital Israelita Albert Einstein, São Paulo, Brazil; Clínica Gastrosaúde, Marília, Brazil; Pontificia Universidade Católica do Paraná (PUCPR), Curitiba, Brazil

**Keywords:** ulcerative colitis, inflammatory bowel diseases, tofacitinib, Janus kinase inhibitors

## Abstract

**Background:**

Ulcerative colitis (UC) is a chronic inflammatory bowel disease which affects the colorectal mucosa with a relapsing–remitting pattern. The therapeutic options currently available for the medical management of UC include many options. Tofacitinib is an oral small molecule, Janus kinase (JAK) inhibitor, more selective for JAK1 and JAK3, which reduces the inflammatory process involved in the pathogenesis of UC.

**Methods:**

Retrospective observational multicentric study of patients with UC who used tofacitinib in any phase of their treatment. Clinical remission and response (according to Mayo score), mucosal healing, primary and secondary loss of response, discontinuation of the drug with possible causes, and the need for dose optimization or switching to biologicals, need for surgery and adverse events were evaluated.

**Results:**

From a total of 56 included patients, clinical remission was observed in 43.6% at week 12, 54.5% at week 26, 57.9% at week 52, and 40% at the last follow-up visit. Clinical response was observed in 71.4%, 81.8%, 89.5%, and 61.8% at the same time periods, respectively. Mucosal healing rates were 50% and 17.8% needed colectomy.

**Conclusions:**

Tofacitinib was effective in induction and maintenance of clinical response and remission rates, compatible to other international real-word studies and meta-analyses.

## Introduction

Ulcerative colitis (UC) is a chronic inflammatory bowel disease (IBD) which affects the colorectal mucosa with a relapsing–remitting pattern that can affect individuals of all ages. The precise etiology of UC is unknown, and curative medical therapy is not yet available. The aim of current pharmacological treatment is to reduce mucosal inflammation inducing and maintaining clinical and endoscopic remission, though these goals are not achieved in all patients.^1,2^

The currently available therapeutic options for the medical management of UC include aminosalicylates, systemic or topical steroids, immunosuppressants (azathioprine, 6-mercaptopurine, and cyclosporine), as well as biological agents such as anti-tumor necrosis factor (anti-TNF) agents (infliximab, adalimumab, and golimumab), anti-adhesion molecules (vedolizumab), and anti-interleukins (ustekinumab).^3^

More recently, therapies with other mechanisms of action targeting different inflammatory pathways were developed and approved for the management of UC. The JAK (Janus kinase) family includes 4 tyrosine kinases: JAK1, JAK2, JAK3, and tyrosine kinase 2 (TYK2).^4^ These enzymes, expressed primarily in hematopoietic cell lines, play a critical role in intracellular cytokine signaling. Binding of a cytokine to its receptor activates receptor-associated JAKs.^5^ The activated JAKs phosphorylate specific tyrosine residues in the cytoplasmic domains of the cytokine receptor subunits, which then act as docking sites for Signal Transducer and Activator of Transcription (STAT) proteins. Activated STATs translocate to the cell nucleus and function as transcription factors to regulate gene transcription in immune cells which are essential in processes such as lymphocyte differentiation, immune regulation, and inflammation.^5^

Tofacitinib is a JAK inhibitor, more selective for JAK1 and JAK3, with an intracellular mechanism of action that blocks DNA transcription and reduces the production of important pro-inflammatory cytokines. Thus, it reduces the inflammatory process involved in the pathogenesis of UC.^6^ Tofacitinib is an oral small molecule, also approved for rheumatoid arthritis, psoriatic arthritis, and ankylosing spondylitis. Randomized phase 3 pivotal studies from the OCTAVE program demonstrated the efficacy and safety profile of tofacitinib in moderate-to-severe UC.^7,8^ More recently, some real-world cohort studies from different parts of the globe and meta-analyses demonstrated the efficacy and safety of tofacitinib in clinical practice, both in patients naive or previously exposed to biological agents.^9–14^ Interestingly, it has been demonstrated that the safety profile of tofacitinib varies widely based on region. For instance, tuberculosis was mostly reported in endemic regions^15^ while the incidence rate of herpes zoster was higher in Japan and Korea as compared to western countries. The reason for such discrepancy is unclear but the possibility of genetic predisposition or different medical practices has been raised. Despite several publications from different countries, there is scarcity of data describing the role of tofacitinib in Latin America.^16,17^ Given that different demographic, socioeconomic, pharmacogenetics, and disease-related factors may exist across populations from different continents, which may limit the generalization of findings, data derived from Latin American patients are warranted. The aim of this study was to analyze the effectiveness and safety profile of tofacitinib in a Brazilian cohort of patients with moderate-to-severe UC.

## Materials and Methods

### Study Design

This was a retrospective, multicentric, and observational study performed in Brazilian patients with moderate-to-severe UC who used tofacitinib in any phase of their treatment, after failure of conventional therapy (aminosalicylates, corticosteroids, and/or immunomodulators) or biological therapy (anti-TNF agents [infliximab, adalimumab, golimumab], vedolizumab, or ustekinumab). The patients were treated at tertiary referral centers in the management of IBD in Brazil. Tofacitinib was approved for UC by the national Brazilian regulatory agency in March 2019.^18^

### Inclusion and Exclusion Criteria

The inclusion criteria were adult patients (over 18 years old) with moderate-to-severe UC (defined as a full Mayo score of 6–12)^19^ treated with tofacitinib for at least 8 weeks, regardless of previous use of other biological agents, on an outpatient basis. The exclusion criteria were patients with other types of colitis (undetermined, microscopic, ischemic, infectious, or Crohn’s colitis), patients with severe UC admitted to the hospital, pediatric and pregnant patients.

### Treatment and Variables Analyzed

Clinical information regarding patients’ demographic characteristics were analyzed, such as sex, age at treatment initiation, smoking status, and disease duration from diagnosis to tofacitinib initiation. Previous medications (corticosteroids and immunomodulators, such as azathioprine and methotrexate), previous use of biological agents (infliximab, adalimumab, golimumab, vedolizumab, or ustekinumab), and disease extension (distal proctitis, left colitis, or extensive colitis) were evaluated, as well as the partial Mayo score before induction and at different time periods. The full Mayo score and endoscopic Mayo subscore at baseline were also evaluated.

After the induction dose of 10 mg of tofacitinib twice daily for 8 weeks and maintenance of 5 mg, patients’ electronic medical records were evaluated. Dose optimization for 10 mg twice daily as maintenance regimen could be used according to physicians’ discretion. Clinical evaluation data were checked at weeks 12, 26, 52, and at the last follow-up by measurement of the partial Mayo score to analyze clinical remission and response. Effectiveness after induction was checked at 12 weeks due to possible limitations associated to data collection in a retrospective observational study (at 8 weeks some patients could not have the post-induction visit due to logistical issues in outpatient consultation scheduling). Patients treated from March 2019 to March 2021 could be included. Data were extracted by investigators and blindly transferred to primary and senior authors. Colonoscopy records were checked to evaluate mucosal healing, at variable times, whenever available. The time of endoscopic assessment (colonoscopy) from tofacitinib induction and the Mayo endoscopic subscore at the first endoscopic examination after treatment initiation was additionally checked. Primary and secondary loss of response to tofacitinib, discontinuation of the drug with possible causes, and the need for dose optimization or switching to biological agents were also analyzed. The need for colectomy during follow-up and the timing of surgery (when present), as well as adverse events (herpes zoster infections, thromboembolic events, upper respiratory tract infections, overall infections, other adverse events, and mortality) during treatment were equally checked.

### Definitions

Clinical remission (primary objective) was analyzed at weeks 12, 26, 52, and the last follow-up, and was defined as a partial Mayo score of less or equal to 2 points.^19^ Specific Mayo subscore additional definitions in clinical remission were not used due to the retrospective observational study design. The last follow-up was defined as the last patient visit or the last date of tofacitinib use, in cases of treatment discontinuation. Clinical response was defined as a reduction of greater or equal to 2 points from baseline on partial Mayo score. Patients were defined as primary nonresponders when presented the same or greater partial Mayo score after 16 weeks. Secondary loss of response was defined as active disease with a partial increase in the Mayo score of 2 or more points after initial response, leading to dose optimization for 10 mg twice daily. Endoscopic remission was defined as an endoscopic Mayo subscore of 0 or 1, according to each endoscopist’s discretion, with no central reading.

### Statistical Analysis

Efficacy data captured in an “as observed” analysis, with denominators comprised only of patients who achieved the different time points of the study and “nonresponder imputation,” where denominators for all time periods comprised the full sample of included patients. Categorical variables were expressed as proportions and compared using chi-square or Fisher’s exact tests, where appropriate. Continuous variables were summarized using mean values, SD, and interquartile ranges. Kaplan–Meier curves were generated for time-to-event data (time until tofacitinib discontinuation in months) and need for colectomy during follow-up. We used IBM SPSS Statistics for Windows, version 20.0 (IBM Corp). The significance level adopted for the statistical tests was 5%.

## Ethical Considerations

This study was approved by the ethics committee of the Pontifical Catholic University of Paraná (PUCPR) and all involved institutions under the reference number 36817920.5.0000.0020. All involved centers had Institutional Review Boards approval. Permission to use data from the IBD databases was granted upon approval by the central institutional review board.

## Results

A total of 56 patients were initially identified and included. Overall, *n* = 56 patients were included for the efficacy analysis at week 12, *n* = 33 at week 26, and *n* = 19 at week 52. Mean follow-up time was 9.8 ± 10.3 months (min. 2 and max. 42). Table 1 describes in detail patients’ demographic and clinical characteristics. As observed, our population was mostly comprised by young patients, nonsmokers, with extensive colitis (Montreal E3), with a median partial Mayo score of 7.06 and Mayo endoscopic subscore of 3. Five patients had concomitant rheumatoid arthritis. Most patients (64.3%, *n* = 38) were previously exposed to anti-TNF agents. A total of 33 (58.9%) used vedolizumab before treatment with tofacitinib and no patients had previous ustekinumab. Overall, 18 patients were naive to biological therapy.

**Table 1.** Baseline characteristics of included patients.

Variable	
Age (years, mean, range)	40.01 (18–70)
Male sex (*n*, %)	29 (51.8)
Disease duration (months, mean, range)	112 (10–372)
Current smoker (*n*, %)	6 (10.7)
Montreal classification (*n*, %)	
E2	13 (23.2)
E3	43 (76.8)
Concurrent rheumatoid arthritis (*n*, %)	5 (8.9)
Previous corticosteroids (*n*, %)	50 (89.3)
Previous azathioprine/6-MP (*n*, %)	36 (64.3)
Previous anti-TNF (*n*, %)	38(67.9)
IFX	29 (51.8)
ADA	3 (5.4)
IFX + ADA	6 (10.7)
Previous vedolizumab (*n*, %)	33 (58.9)
Partial Mayo score (mean ± SD, min–max)	7.06 ± 1.38 (3–9)
Mayo endoscopic subscore (*n*, %)	
2	18 (32.1)
3	38 (67.9)

Abbreviations: ADA, adalimumab; anti-TNF, anti-tumor necrosis factor; IFX, infliximab.

**Figure 1.**
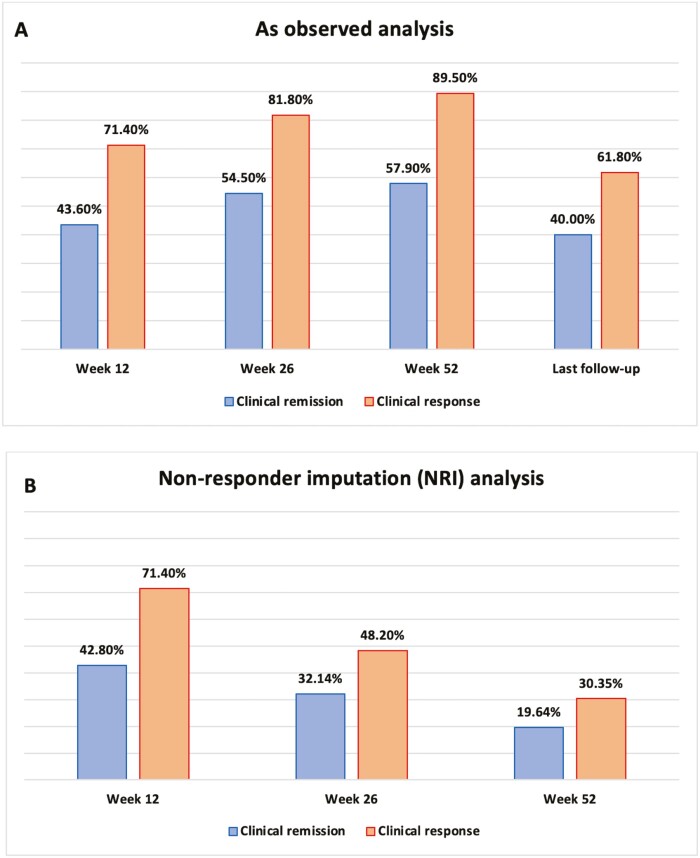
Efficacy data (clinical remission and response to tofacitinib) at different time periods. (A) As observed analysis. (B) Nonresponder imputation analysis.

Regarding the primary outcome of the study, in “as observed” analysis, clinical remission was observed in 43.6% of the patients at week 12, 54.5% at week 26, 57.9% at week 52, and 40% at the last follow-up visit. Clinical response was observed in 71.4%, 81.8%, 89.5%, and 61.8% at the same time periods, respectively. In a nonresponder imputation analysis, using 56 patients as denominators for all time periods, clinical remission was observed in 42.8% at week 12, 32.14% at week 26, and 19.64% at week 52. Clinical response was observed in 71.4%, 48.2%, and 30.35% of the 56 patients, in the same time periods, respectively. These data are illustrated in Figure 1A and B.

When stratified according to previous exposure to biologics, there were no significant differences regarding clinical response or remission. These data are illustrated in Supplementary Table S1.

Overall, 12/56 (21.4%) patients were considered as primary nonresponders to tofacitinib. Secondary nonresponse was observed in 11/56 (19.6%) patients. Optimization to 10 mg twice daily, as maintenance therapy, was performed in 15/56 (26.8%) patients between weeks 26 and 52, and discontinuation of tofacitinib occurred in 19/56 (33.9%), due to lack of efficacy (*n* = 8), need for colectomy (*n* = 10), and adverse events (*n* = 1). Eleven patients (19.6%) switched tofacitinib for biological therapy (7 to ustekinumab, 3 to vedolizumab, and 1 to infliximab).

A total of 32 patients underwent a control endoscopic examination after tofacitinib initiation, in a mean period of 7.69 ± 4.54 (min. 1 and max. 24) months. Mucosal healing was observed in 16 patients (50%), 4 with a Mayo endoscopic subscore of 0 and 12 with a subscore of 1.

**Figure 2.**
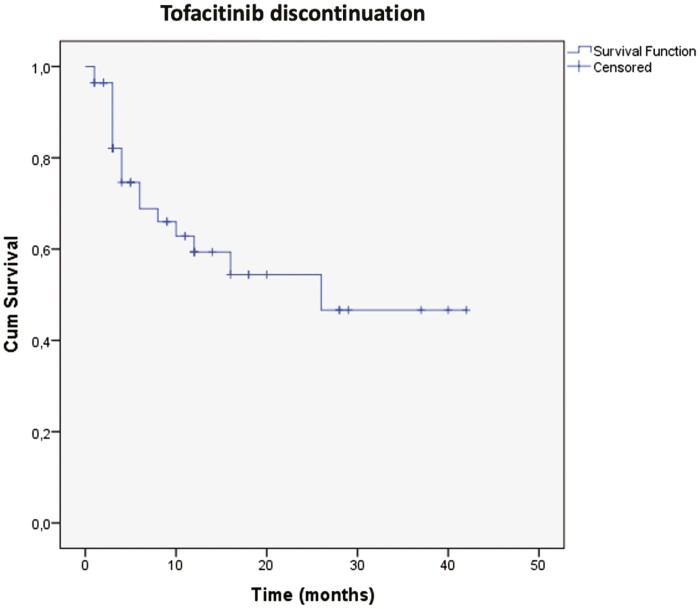
Kaplan–Meier curve with time to tofacitinib discontinuation as main event.

**Figure 3.**
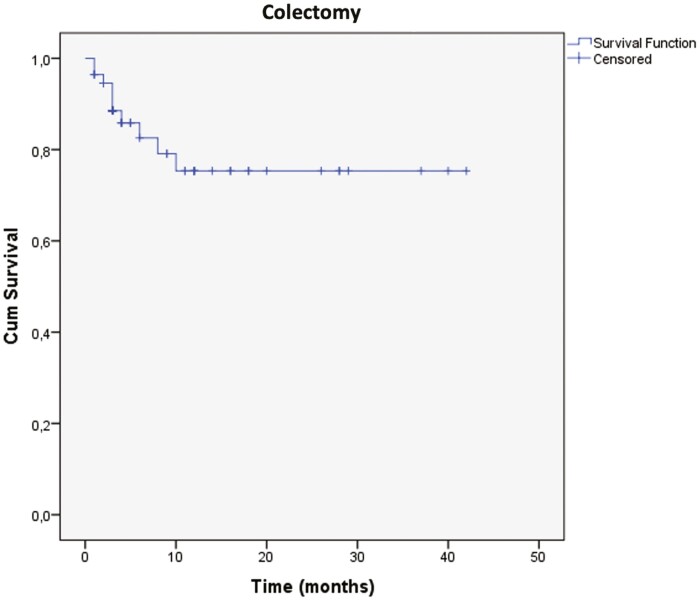
Kaplan–Meier curve with time to colectomy as main event.

Figure 2 describes the persistence with tofacitinib treatment over time in our cohort (time-to-event considering discontinuation of the drug). Mean total time of use of tofacitinib was 24.47 ± 3.02 months (95% CI, 18.54–30.4). Figure 3 describes the Kaplan–Meier curve of colectomy-free survival. Mean time to colectomy was 32.85 ± 2.62 months (95% CI, 27.72–37.99). From the 10 patients undergoing surgery, 6 were submitted to 3-stage colectomy (total colectomy and ileostomy first) and 4 underwent a 2-stage procedure (restorative proctocolectomy with pouch and loop ileostomy).

Regarding safety, Table 2 describes in detail the main adverse events observed in our cohort. Overall, 19 patients (33.9%) developed an adverse event. The most common was upper respiratory tract infection (*n* = 5). Thromboembolic events occurred in 2 patients (both with deep vein thrombosis). Only 1 patient developed herpes zoster infection and 1 patient had COVID-19 during treatment with tofacitinib. One patient died during treatment, a 54-year-old lady, due to acute myocardial infarction confirmed by autopsy.

**Table 2.** Adverse events observed in 19/56 patients.

Adverse event	*N* (%)
Upper respiratory tract infection	5 (8.9)
Thromboembolic events	2 (3.6)
*Clostridioides difficile* infection	1 (1.8)
Urinary tract infection	1 (1.8)
Herpes zoster	1 (1.8)
Other infectious events	4 (7.1)
Gastroenteritis	1 (1.8)
CMV infection	1 (1.8)
Parotiditis	1 (1.8)
COVID-19	1 (1.8)
Other adverse events	9 (16.1)
Anemia	4 (7.1)
Dyslipidemia	3 (5.4)
Acute myocardial infarction	1 (1.8)
Liver enzymes alterations	1 (1.8)
Death	1 (1.8)

Abbreviation: CMV, cytomegalovirus.

## Discussion

In this multicentric Brazilian study, tofacitinib was effective in achieving clinical response and remission as induction and maintenance therapy, with a similar safety profile to what was described in other similar real-world observational studies. Clinical remission after 1 year of treatment occurred in 57.9% and clinical response was observed in 89.5% of patients who could persist under treatment for this period. Rates of secondary loss of response, dose optimization, and drug discontinuation, as well as colectomy, were also comparable with previous studies with similar methodology.^9–14^

Our population of patients was mostly comprised by patients with previous exposure to anti-TNF agents (38/56—67.9%) and longer disease duration (mean 112 months, almost 10 years since diagnosis of UC), what is compatible with other real-world studies with tofacitinib.^9–14^ In the OCTAVE trials, nearly half of the patients were naive to biological agents.^7,8^ The Brazilian label includes the indication for patients who are naive to biological therapy, and 18/56 (32.1%) of our patients were not previously exposed to anti-TNF agents. This is a slightly larger proportion than other international cohorts, which predominantly analyzed patients who had previously failed to other biologics. In a meta-analysis by Taxonera et al, which included 17 real-world studies with tofacitinib in UC, 88.4% of the 793 included patients had been previously exposed to biologics.^12^ Only 11.6% were naive to biological therapy. In another meta-analysis with 9 studies, 91% of the 830 patients were previously treated with anti-TNF agents.^10^ Our slightly larger proportion of naive patients is one of the most important characteristics of the present study.

At induction (8 weeks), the meta-analysis from Lucaciu et al described clinical response in 51% (95% CI, 41%–60%) and remission in 37% (95% CI, 26%–45%) of included patients.^10^ Authors also demonstrated that after a median follow-up of 24 weeks, these numbers dropped to 40% (95% CI, 31%–50%) and 29% (95% CI, 23%–36%), respectively. Our rates of response and remission in induction after 12 weeks (71.4% and 43.6%) and after 26 weeks (81.8% and 54.5%) were slightly higher than those previously described in this meta-analysis. Although there is significant heterogeneity in methodology and clinical scoring systems among the available studies, which limits extensive comparisons with our results, a possible reason for these findings is the less clinically refractory population of our cohort.

In the longer term, clinical response was described in 42% and clinical remission in 41% of included patients, in the larger meta-analysis of real-world cohorts, after 52 weeks.^12^ In the same period (1 year), efficacy results of our study showed higher rates of clinical response (89.5%) and clinical remission (57.9%). Our numbers at last follow-up were similar to those described by Taxonera et al (clinical response of 61.8% and clinical remission of 40%).^12^ This can be explained by the fact that 90% of the patients in this meta-analysis were biologic-experienced and approximately two-thirds had previously failed both an anti-TNF agent and vedolizumab. An additional reason could be based in the type of analysis performed in our study. The “*as observed*” analysis uses as denominators only patients who reached the preestablished time periods using tofacitinib. This would bias numbers as only responders could persist using the agent, putting response and remission proportions in a higher scale. In our cohort, From the 19 patients in remission at week 52, 57.9% were females, 78.9% had extensive colitis, 68.3% had previous anti-TNF, and 52.6% previously used vedolizumab. If these characteristics comprise possible factors associated to remission, this should be analyzed in a larger sample of patients, with multivariate analysis, to reduce bias.

Mucosal healing was observed in 16/32 (50%) patients who underwent endoscopic examinations after treatment initiation, in a mean period of 32.85 ± 2.62 months (95% CI, 27.72–37.99). Data after induction (12–16 weeks) revealed 35.1% of mucosal healing.^12^ Our rates are possibly higher due to our less stringent definition of mucosal healing. Nonetheless, it is worth mentioning that this was the same definition used in the OCTAVE trials.

There is still some controversy in real-world studies if there are differences in the effectiveness of tofacitinib regarding the previous exposure to biological agents’ status. In our cohort, when the population was stratified according to previous exposure to biologics, there were no significant differences regarding clinical response or remission (Supplementary Table S1). These findings were similar to what was reported in a study from the United Kingdom, which included 119 patients.^14^ Data from the OCTAVE trials also demonstrated that the treatment effects were similar between those who had received previous treatment with a TNF antagonist and those who had not.^7^ However, deltas between patients in the treatment arms and placebo were quite similar.^7,8^ Thus, there is still a substantial knowledge gap on the performance of tofacitinib in naive patients in clinical practice.

Treatment discontinuation with tofacitinib was observed in 33.9% of our included patients, due to lack of efficacy in 42.1%, need for colectomy in 52.63%, and adverse events in only 5.2%. Eleven patients (19.6%) switched from tofacitinib to biological therapy. Lucaciu et al, in a meta-analysis, demonstrated a similar discontinuation rate of tofacitinib as our study’s (35%), mostly due to loss of response (51%), colectomy (19%), and adverse events (20%).^10^ Median drug persistence with tofacitinib was 24.47 ± 3.02 months (95% CI, 18.54–30.4), what was compatible to other real-life studies. Our rate of primary nonresponders (21.4%), in contrary, was lower as what was observed in this pooled analysis (51%),^10^ despite similar to findings from the multicentric experience from the United Kingdom (29%).^14^ This difference could be based in definitions of primary nonresponse in different studies, which were included in the meta-analysis.

Colectomy rates are decreasing in the biologic era according to recent population-based studies.^20^ In our cohort, 10 out of 56 patients (17.85%) underwent colectomy during treatment with tofacitinib, with a mean time to surgery of 32.85 ± 2.62 months (95% CI, 27.72–37.99). Again, our numbers were compatible to what was described in previous cohort studies and meta-analyses of real-world data. A study from the United States reported a need for colectomy of 5.6%, and the aforementioned multicentric UK study, of only 3.7%.^9,14^ The 2 meta-analyses performed in real-world cohort studies had rates of 9%–13%.^10,12^

In the present study, the safety profile of tofacitinib was consistent with what was observed in most pivotal studies and safety subanalyses.^7,8^ The upper respiratory tract infections were the most prevalent adverse events. One death was observed due to complications after acute myocardial infarction. However, according to the treating physician, no correlation with tofacitinib use could be identified.^16^ Only 1 case of herpes zoster infection was observed. With longer follow-up, more cases could be captured in our cohort, similarly to long term studies.^21,22^ No malignancies were observed.

Our study is associated with some inherent limitations. First, typical methodological issues from retrospective multicentric studies, which involve data capturing from electronic medical records, could be associated to bias. Although most involved units follow the ECCO guidelines, different physicians were involved in patient care, with possible variability in clinical practice. The relatively short period of follow-up could also have contributed to variation in our results. Despite these limitations, the precise definitions of analyzed variables may give an adequate overall snapshot of what is observed in daily clinical practice with new treatment options in our country. Our study represents to date the first detailed experience with tofacitinib for the management of UC in Latin American patients.

## Conclusions

In summary, our Brazilian multicentric observational study demonstrated clinical response and remission rates which are compatible to other international real-world studies and meta-analyses. The safety profile, mucosal healing and colectomy rates were also in tune with what is described in pivotal trials and previous cohort descriptions. More evidence is needed in order to position tofacitinib more precisely in Latin American treatment algorithms.

## Supplementary Data

Supplementary data is available at *Crohn’s and Colitis 360* online.

Supplementary Table S1. Clinical remission and response rates. Considered denominators were the number of patients in remission or response in different time points.

otac050_suppl_Supplementary_Table

## Data Availability

The study full data are available upon request and approval of central IRB.
