# Supplementary material for: Effectiveness and Safety of Tofacitinib in the Management of Ulcerative Colitis: A Brazilian Observational Multicentric Study
Source: Crohns Colitis 360. 2022 Dec 19;5(1):otac050. doi: 10.1093/crocol/otac050 (PMC9855307; doi:10.1093/crocol/otac050)
Supplement: otac050_suppl_Supplementary_Table [file otac050_suppl_supplementary_table.docx]

|  | **Anti-TNF naïve (n=18)** | **Anti-TNF exposed (n=37)** | **P value** |
| --- | --- | --- | --- |
| **Clinical remission (%)** | | | |
| **Week 12 (n=24)** | 11 (45.8) | 13 (54.2) | 0.683 |
| **Week 26 (n=18)** | 10 (55.6) | 8 (44.4) | 0.804 |
| **Week 52 (n=11)** | 3 (27.3) | 8 (72.7) | 0.216 |
| **Clinical response (%)** | | | |
| **Week 12 (n=40)** | 15 (37.5) | 25 (62.5) | 0.335 |
| **Week 26 (n=27)** | 10 (37.1) | 17 (62.9) | 0.637 |
| **Week 52 (n=17)** | 5 (29.4) | 12 (70.6) | 1.000 |

**Supplementary Table 1: clinical remission and response rates. Considered denominators were the number of patients in remission or response in different time points.**
